# Power Spectral Density Evaluation of Laser Milled Surfaces

**DOI:** 10.3390/ma11010050

**Published:** 2017-12-29

**Authors:** Raoul-Amadeus Lorbeer, Jan Pastow, Michael Sawannia, Peter Klinkenberg, Daniel Johannes Förster, Hans-Albert Eckel

**Affiliations:** 1 German Aerospace Center (DLR), Institute of Technical Physics, Pfaffenwaldring 38-40, 70569 Stuttgart, Germany; Jan.Pastow@de.TRUMPF.com (J.P.); michael.sawannia@ifsw.uni-stuttgart.de (M.S.); peter.klinkenberg@bosch.com (P.K.); Hans-Albert.Eckel@dlr.de (H.-A.E.); 2 Institut für Strahlwerkzeuge, University of Stuttgart, Pfaffenwaldring 43, 70569 Stuttgart, Germany; daniel.foerster@ifsw.uni-stuttgart.de

**Keywords:** surface roughness, surface unevenness, laser milling, power spectral density, micro crystalline, Hall-Petch, EBSD

## Abstract

Ablating surfaces with a pulsed laser system in milling processes often leads to surface changes depending on the milling depth. Especially if a constant surface roughness and evenness is essential to the process, structural degradation may advance until the process fails. The process investigated is the generation of precise thrust by laser ablation. Here, it is essential to predict or rather control the evolution of the surfaces roughness. Laser ablative milling with a short pulse laser system in vacuum (≈1 Pa) were performed over depths of several 10 µm documenting the evolution of surface roughness and unevenness with a white light interference microscope. Power spectral density analysis of the generated surface data reveals a strong influence of the crystalline structure of the solid. Furthermore, it was possible to demonstrate that this effect could be suppressed for gold.

## 1. Introduction

Laser milling is a process whose results are altered dramatically by a multitude of parameters. The basic ablation effects on metals in vacuum can be categorized by phase transitions such as, e.g., melting, vaporization, spallation or phase explosion [1], depending on the applied fluence and pulse duration as basic parameters of system engineering [2,3,4]. Nevertheless, the change of surface roughness during processing has a variety of influencing factors. On the one hand, the design of the optical system with the spatial overlap as a process parameter can influence the roughness [2,4]. In addition, the delay between successive pulses (pulse repetition rate) plays an important role with regard to the so-called heat accumulation, which in turn can directly influence the roughness and alter the processing result [4,5,6]. Interestingly, these very same effects would lead to a significant degradation of laser ablative thrusters [7]. Thrusters which bear the potential of generating a very precise thrust for scientific missions such as, e.g., the LISA mission, a mission with the aim to detect gravitational waves which might give testimony of the very beginning of our universe [8,9].

This shortened story connecting the beginning of the universe to laser milling applications is dictated by the investigation of a thruster concept called MICROLAS [1,10]. The MICROLAS concept utilizes the properties of the very hot excavated material from the laser ablative process. This material can be heated to plasma temperatures of several 10,000 K which leads to very high exhaust speeds due to the expansion of the plasma [11]. High exhaust speeds in general are very desirable for a prolonged thruster utilization. The necessary amount of momentum and thrust can be generated by adding up the correct amount and rate of ablation events respectively. Each event adds a tiny momentum in the range of 1 nNs allowing for a subtle momentum control [1,12]. With non-mechanical beam steering as foreseen in the MICROLAS concept [1,10] this would be the only source of disturbance, which in the case of LISA potentially allows for a positioning accuracy of the satellites below 10 nm [9].

Since the laser ablation or rather the laser milling process itself is the dominant source of error to the thrust generation, runaway effects in laser milling can lead to thrust degradation and mission failure. In a previous publication [7], surface roughness and surface unevenness were identified as two independent candidates for thrust degradation. Nevertheless, the necessary data on the evolution of these two surface parameters while ablating material under vacuum conditions was missing. Therefore, experiments investigating the surface roughness after milling to depths of several 10 µm utilizing a 500 ps short pulse laser were performed. In contrast to prior work now varying surface roughness and surface unevenness are distinguished. Both properties are likely to reduce thrust quality. To separate between both regimes the method of power spectral density (PSD) evaluation was chosen which can be applied to surface profiles [13,14]. Tested materials were aluminum, copper, gold, and graphite.

## 2. Materials and Methods

### 2.1. Experiments

Our setup for milling experiments has been described previously [7,15]. A laser scanner with attached f-theta objective was used to focus the circular polarized laser light of a TEEM Photonics 500 ps microchip laser emitting at 1064 nm laser wavelength onto the sample surface. A maximum of 82.5 µJ of pulse energy and 1000 pulses per second were available from the system. The focal spot was slightly oval and had an average radius $\omega_{0}$ of 17±2µm. This allows to calculate the peak fluence $F_{\mathrm{peak}}$ of a Gaussian shaped laser spot. Spot scanning was performed with a galvo scan head (intelliSCAN 14, Scanlab, Puchheim, Germany) and a f-theta objective (f = 167 mm). The sample was placed within a vacuum chamber and two anti-reflective coated windows were placed between the sample and the laser scanning system. One window sealed off the air to the vacuum, the second window shielded the sealing window from excavated material, which generated a sputtered layer over time reducing the transmittivity of the window. Therefore, the second window was exchanged when it’s transmittivity did reduce laser pulse energies at the target below the desired amount for the following ablation sequence.

The milling process itself consisted of the scanning of an area 1 mm × 1 mm in size under 18 different line scanning directions each rotated by 10°, also called hatching angles. Due to laser synchronization with the scanner system a constant spot displacement of 12 µm (corresponding to a scan velocity of 12 mm/s) and a constant line displacement of 12 µm was achieved. After accomplishing a rotation of 180° (18 single area scans), the procedure was repeated such that at a fixed laser pulse energy 1, 2, 4, 8, 16 and 32 repetitions of the hatching pattern were performed. This procedure allows to compare different milling depths under almost identical conditions. The lowest pulse energies used in the processes were chosen to be slightly higher than the ablation threshold of the material.

**Figure 1:**
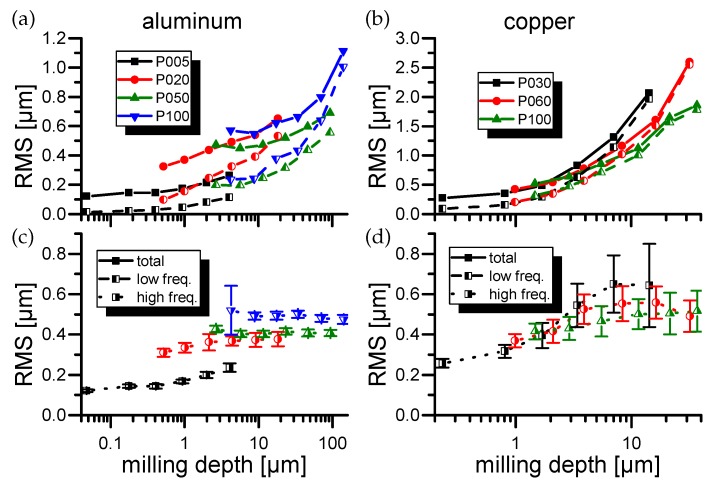
(**a**,**c**) Root mean square (RMS) roughness of aluminum as well as RMS roughness for low and high frequencies. ($P005\;\hat{=}\;3.2\pm 0.1\;µJ$ or $F_{\mathrm{peak}}$ = 0.70±0.19J/cm${}^{2}$, $P020\;\hat{=}\;12.5\;\pm 0.2\;µJ$ or $F_{\mathrm{peak}}$ = 2.75±0.69J/cm${}^{2}$, $P050\;\hat{=}\;31.7\pm 0.5\;µJ$ or $F_{\mathrm{peak}}$ = 0.70±0.19J/cm${}^{2}$, $P100\;\hat{=}\;63\;\pm 1\;µJ$ or $F_{\mathrm{peak}}$ = 13.88±3.49J/cm${}^{2}$). (**b**,**d**) RMS roughness of copper as well as RMS roughness for low and high frequencies. ($P030\;\hat{=}\;19.7\pm 0.1\;µJ$ or $F_{\mathrm{peak}}$ = 4.34±1.04J/cm${}^{2}$, $P060\;\hat{=}\;33.5\pm 6\;µJ$ or $F_{\mathrm{peak}}$ = 7.38±3.06J/cm${}^{2}$, $P100\;\hat{=}\;55\pm 10\;µJ$ or $F_{\mathrm{peak}}$ = 12.2±5.05J/cm${}^{2}$).

**Figure 2:**
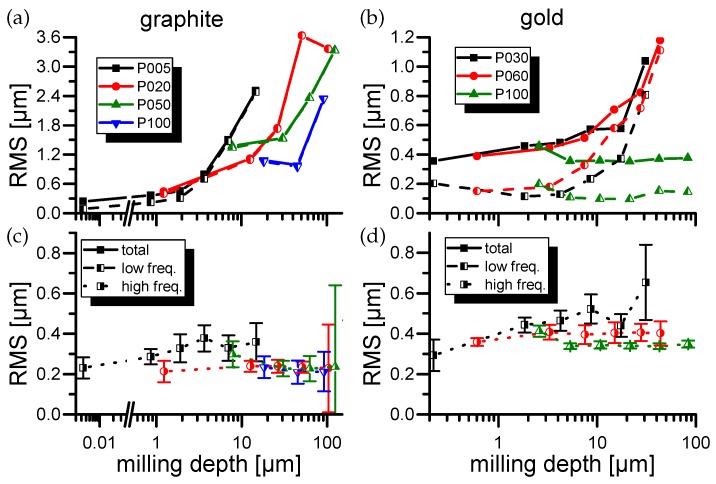
(**a**,**c**): RMS roughness of graphite as well as RMS roughness for low and high frequencies. Low frequency RMS graphs are obscuring the total RMS graphs due to similar values. Milling depths above 150 µm were omitted due to rim artifacts for high milling depths. ($P005\;\hat{=}\;2.8\pm 0.1\;µJ$ or $F_{\mathrm{peak}}$ = 0.62±0.17 J/cm${}^{2}$, $P020\;\hat{=}\;10.7\pm 0.3\;µJ$ or $F_{\mathrm{peak}}$ = 2.36±0.62J/cm${}^{2}$, $P050\;\hat{=}\;29.4\;\pm 1.3\;µJ$ or $F_{\mathrm{peak}}$ = 6.48±1.81J/cm${}^{2}$, $P100\;\hat{=}\;58\pm 3\;µJ$) or $F_{\mathrm{peak}}$ = 12.78±3.67J/cm${}^{2}$. (**b**,**d**): RMS roughness of gold as well as RMS roughness for low and high frequencies. ($P030\;\hat{=}\;15.7\pm 2.5\;µJ$ or $F_{\mathrm{peak}}$ = 3.46±1.36J/cm${}^{2}$, $P060\;\hat{=}\;39\;\pm 6\;µJ$ or $F_{\mathrm{peak}}$ = 8.59±3.34J/cm${}^{2}$, $P100\;\hat{=}\;52\;\pm 9\;µJ$ or $F_{\mathrm{peak}}$ = 11.45±4.68J/cm${}^{2}$).

The remaining laser intensity within the chamber was measured after every opening of the vacuum chamber to compensate for any material deposition on the shielding window. Nevertheless, material deposition led to uncertainties in the overall applied pulse energies as depicted in the captions of Figure 1 and Figure 2.

Gold, copper and aluminum were obtained as mirror polished rectangular plates with a typical remaining surface roughness below 10 nm (Kugler, Salem, Germany). Copper and aluminum had a surface area of 25 mm × 25 mm whereas the gold sample had a surface area of 20 mm × 20 mm. The pyrolytic graphite was purchased as cuboid 25 mm × 25 mm × 3 mm in size (Dreamtime24, Munich, Germany). The sample was polished by hand with sand paper first with a grain of 2400 and at last 4000. The remaining surface standard deviation typically remained below 300 nm.

### 2.2. Data Acquisition

After laser irradiation, the surfaces of the generated cavities were scanned with an optical white light interferometer (Veeco NT9100, Munich, Germany) microscope. To optimize light collection efficiency and therefore generate as many depth points as possible the objective with the highest numerical aperture (NA≈0.5) was chosen. To scan entire cavities the depth images were captured in an automated manner and stitched by a custom FIJI (ImageJ) macro based on the “Grid/Collection stitching” plugin [16].

Exemplary SEM images are appended as Supplementary Figures S1–S4.

### 2.3. Power Spectral Density Evaluation

**Figure 3:**
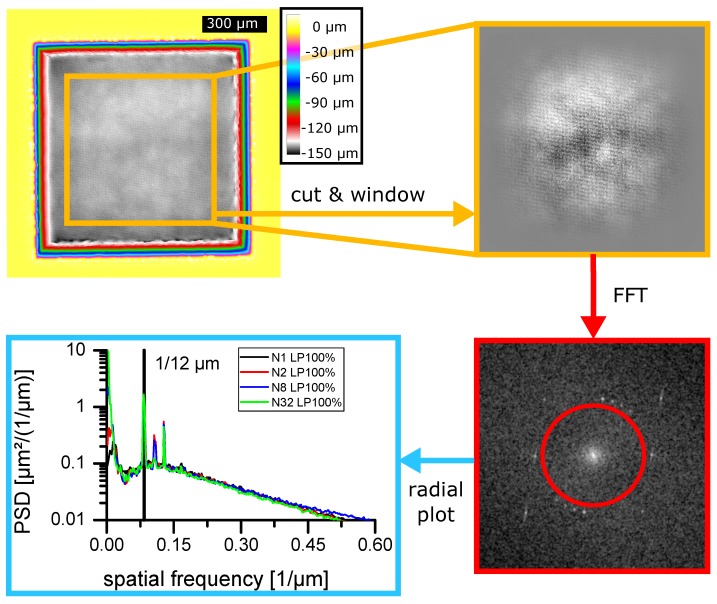
Scheme of power spectral densities (PSD) calculation. Data was taken from aluminum sample for 32 hatching patterns and a pulse energy of 63±1µJ ($F_{\mathrm{peak}}$ = 13.88±3.49J/cm${}^{2}$ at 100% laser power). **Orange box**: Surface profile data is cropped to the region of interest. **Red box**: After windowing a FFT is performed resulting in power spectral density. The red circle indicates the chosen border between low and high frequencies. **Blue box**: PSD result after correct normalization and radial binning. The vertical line indicates the laser spot pattern frequency (112µm).

Depth images of the cavities were processed by analyzing their power spectral densities (PSD). The procedure can be separated into four stages (compare Figure 3):

1. Selecting a region of interest (ROI) within the cavities. Due to the supported resolution a ROI size of 2048 × 2048 pixels was chosen. Then the image was cropped to the ROI specifications. (compare Figure 3 top left)
2. The average value of the new image was subtracted from each value. Afterwards, a PSD conservative (introducing a factor of 43) weighting function reproducing a 2-dimensional Hanning window H(x,y) was applied. (compare Figure 3 orange box)
  $$H\left(x,y\right)=\frac{4}{3}\cdot \left(1+\sin\left(2\pi \frac{x-\underline{x}}{\bar{x}-\underline{x}}\right)\right)\cdot \left(1+\sin\left(2\pi \frac{y-\underline{y}}{\bar{y}-\underline{y}}\right)\right)\;\;\;,$$
  with $\bar{x},\underline{x},\;\bar{y},\underline{y}$ as the range of positions of the cropped image and x,y as image coordinates.
3. A fast Fourier transformation (FFT) was applied and the square of the absolute value at each frequency was computed. (compare Figure 3 red box)
4. The resulting spectra were summed up to neglect direction-dependent information. Here, two variants came to use.
  (a) All wave vectors corresponding to the same frequency were put into the same “bucket”. This was done using the ImageJ “Radial Profile Angle” plugin [17]. (compare Figure 3 blue box)
  (b) The values within a previously defined circle/frequency range were summed resulting in the “low frequency” roughness as well as all values outside the circle/frequency range resulting in the “high frequency” roughness. (compare Figure 3 red circle)

The algorithm was implemented using ImageJ-Macro language. Scaling of the generated pixel frequencies to physical correct values was achieved by calculating the corresponding scaling factor

$$S=\left(2\pi \cdot r_{px}\cdot \Delta r_{px}\right)\;\cdot \;\frac{\Delta r_{px}}{{\bar{r}}_{µm}}\;\cdot \;\frac{1}{n_{x}^{2}\cdot n_{y}^{2}}\;\;\;.$$

*S* consists of three terms. The first term removes the averaging of the “Radial Profile Angle” plugin by approximating the averaging process with the corresponding circle with radius $r_{px}$ and bin size $\Delta r_{px}$ in pixel scaling. The second term converts from pixel scaling to frequency scaling with ${\bar{r}}_{µm}$ defining the border length of the evaluated area in µm. Finally, a re-normalization with $\frac{1}{n_{x}^{2}\cdot n_{y}^{2}}$, where $n_{x}$ accounts for the number of pixels in *x*-direction and $n_{y}$ for the y-direction respectively, was necessary to compensate for ImageJ-FFT specific scaling effects.

This procedure results in a list of values whose numerical integration leads to the variance of the surface

$$\sigma_{\underline{k}\ldots \bar{k}}^{2}=\int_{\underline{k}}^{\bar{k}}dk\;\mathrm{PSD}\left(k\right)\;\;\;,$$

where $\underline{k}$ and $\bar{k}$ can be chosen to reproduce the frequency ranges depicted as “high frequency” (HF) and “low frequency” (LF) in Section 3.1. In the following the square root of the variance, omitting the zero frequency, will be referred to as root mean square (RMS) value.

### 2.4. Power Spectral Density Variation

To indicate variations of the surface roughness, which can introduce thrust noise [7], a third processing step was added:

1. A fast Fourier transformation was applied to the complete profile data set and the low frequencies of the spectrum were removed (compare Section 2.3 step (4b)).
2. An inverse fast Fourier transformation was applied resulting in an image containing only “high frequency” surface modulations.
3. An identical ROI to the previous methods was chosen and the image was cropped to the ROIs size.
4. The image was split into 16 × 16 sub-/sample-images. Standard deviation of every sample-image was calculated and finally the standard deviation of the surfaces standard deviations was taken as an estimate for the uncertainty of HF-STD or rather surface roughness.

### 2.5. Electron Backscattered Diffraction (EBSD) Analysis

An Electron Backscattered Diffraction (EBSD) analysis was performed with the gold sample.

EBSD combines both methods in a modified scanning electron microscope. Bragg scattering of surface penetrating electrons is recorded and related to the current scanning position, which allows to identify crystal orientations with high spatial resolution.

The sample was prepared by vibration polishing, depleting several µm of the surface to ensure imaging of the underlying crystal structure unaffected by the laser milling process. The measurements were performed at the Materials Testing Institute University of Stuttgart (MPA Stuttgart) with an Auriga SEM (ZEISS, Jena, Germany) and the TSL EBSD (EDAX, Mahwah, NJ, USA) unit.

## 3. Results

### 3.1. Roughness Spectra

Figure 3 (blue box) displays the radial distribution of noise over the spectrum of surface disturbances for aluminum at the maximum laser intensity used in the experiment. Several aspects which allow to differ between “low” and “high” frequencies can be seen in the diagram. For low ablation depths as, e.g., N1 corresponding to one application of a hatching pattern, the PSD values stay well below 1µm21µm. The more the patterns are applied and respectively the more material is ablated, the more the PSD changes. The most eminent changes appear at “low” frequencies below $0.06\;\frac{1}{µm}$ while the higher frequencies are evidently only subject to minor changes. By using this type of representation (Figure 3 (blue box)) all laser milled cavities were examined and a separation between “high” and “low” at $0.07\;\frac{1}{µm}$ directly below the first peak in the spectrum was chosen. The first peak itself has the same frequency as the chosen spot displacement pattern of $\frac{1}{12\;µm}=0.08\bar{3}\frac{1}{µm}$.

A more detailed study of the red box in Figure 3 reveals that in the case of 32 repetitions with the highest available laser energy on aluminum, the first peak does not appear to result from all hatching directions. Starting at the last hatching angle going backwards, the amplitude of the spectral peak decreases successively.

### 3.2. Roughness Measurements

The RMS roughness results for all four samples are displayed in Figure 1 and Figure 2. First of all, it can be seen that generally the “total” RMS roughness rises for deeper milling depths at constant laser milling parameters. By observing the surface profiles by bare human eye it can be seen that rough structures, which especially for copper show strong similarities with the granular structure of the material, dominate the surface RMS. Especially at fluences close to the ablation threshold, this leads to the erosion of “sharp and steep” edges strongly influencing the HF-RMS values and their standard deviation (compare Figure 1 black high freq. curve).

The separation into “low” and “high” frequencies allows to separate the overall surface unevenness represented by low frequency RMS (LF-RMS) from the individual laser spot roughness represented by high frequency RMS (HF-RMS). Consistently for all materials the LF-RMS show a trend similar to the total RMS values whereas the HF-RMS values tend to saturate within a few milling cycles.

The HF-RMS of aluminum rises with higher laser intensities in contradiction to the other three samples which show improving (lower) HF-RMS values with higher laser powers. Especially, pyrolytic graphite drops below the initial surface roughness after the manual polishing progress for all laser powers but the lowest.

Furthermore, within the group of HF-RMS values, the results generated with the strongly asymmetric crystalline pyrolytic graphite (Figure 2) show the lowest roughness values. Here again low fluences lead to erosion effects. In the case of pyrolytic graphite several randomly distributed spikes range from the surface down to the current cavity depth. This leads to an inconsistent roughness error in the HF regime (compare Figure 2 black high freq. curve).

In the data obtained from the gold experiments an anomaly can be observed. For the highest laser power the total RMS of gold stays consistently low. The generated roughness consists dominantly of HF-RMS components.

**Figure 4:**
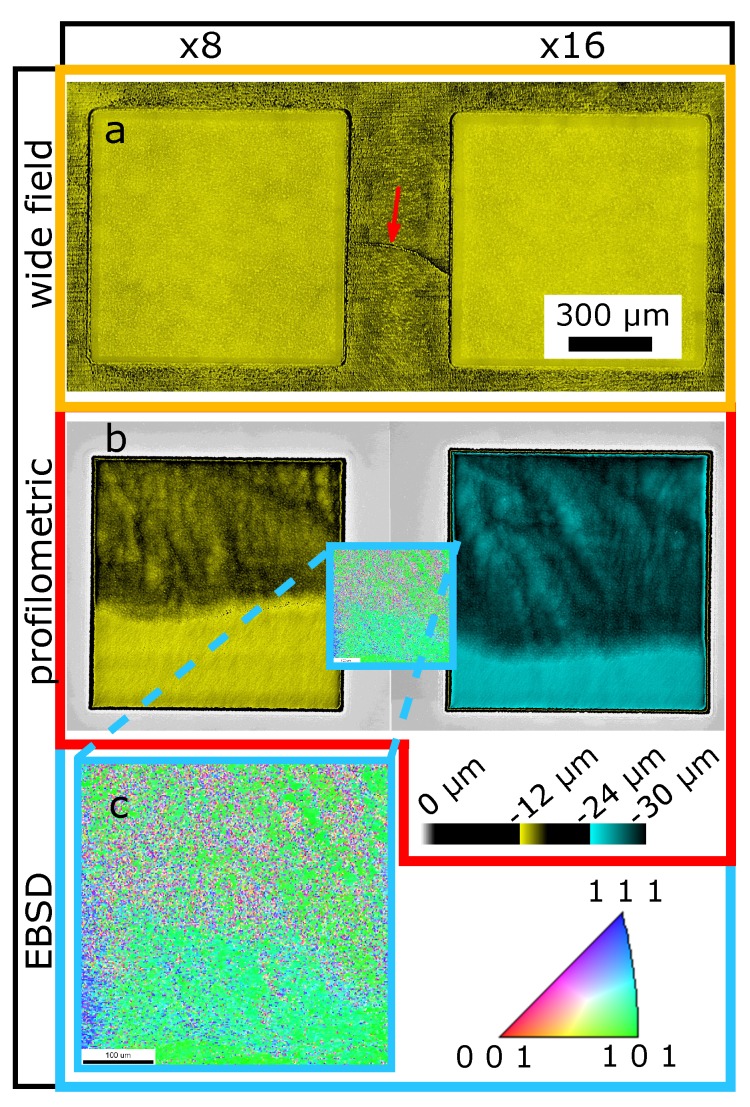
Cavities P060_C8 and P060_C16 (39±6µJ with 8 and 16 hatching patterns) in gold, with intermediate area. (**a**) Wide field microscopy image. Red arrow indicates crack. (**b**) Surface profile images. (**c**) Electron Backscattered Diffraction (EBSD) image.

Further investigation of this effect lead to the results presented in Figure 4. The graphic shows cavities from the P060 (60% laser power) group. Here, the effect of low unevenness sets in. It actually correlates with a crack visible under a wide field microscope (red arrow Figure 4a). The profilometric images (Figure 4b) show the difference in surface structure separating the cavities in a top and bottom part. The bottom side represents the quality of the cavities generated at the highest laser power. This information indicates that the effect could be introduced by the material itself. Therefore, an EBSD analysis was performed as shown in Figure 4 (blue box). In this view, two types of crystalline structures correlated to the different areas can be identified. The top area showing higher unevenness correlates to areas with micro-crystalline structures of varying densities. The bottom area shows significantly less micro-crystalline structures. Especially crystal orientations towards the (0 0 1)-plane are strongly reduced.

The microcrystalline structures were only visible after removing material to an approximate depth of 5 µm from the surface by vibration polishing.

## 4. Discussion

The core question will now be discussed: Can surface roughness as a source for thrust noise in the MICROLAS concept be omitted? The short answer might be yes. Ideal surface evolution in some of the gold measurements (compare Figure 2) was observed. These showed that indeed it is possible to have minor evolution for both surface roughness and unevenness. Even to the point that the surface roughness is constant over time and space (scan position).

Despite this direct observation the evaluation by PSD analysis and the separation into HF- and LF-RMS allowed to analyze both aspects of unevenness and roughness separately. To start with the surface roughness, a consistent saturation effect limiting the roughness to several 100 nm in all cases was shown.

With linear models this effect is not to be expected [7]. This means that the roughness imprinted onto the surface by the scanning pattern saturates as well. In the example of Figure 3, one can deduce that for the set parameters only the last four hatches contribute to the surface roughness. Furthermore, only the lines and not the neighboring spots do generate a pattern indicated by the missing points in horizontal directions.

The measurements with parameters close to the ablation thresholds of the materials show differences in the certainty of the HF-RMS values. Especially copper and graphite deviate strongly. The most likely explanation might be erosion effects, since in the profile images it becomes clear that copper creates steeper edges at crystal borders and graphite expresses several spikes not remaining after the ablation process.

Those two materials exhibit a stronger variation of surface roughness in general. Even though the uncertainty is lower at higher pulse energies, they are bigger compared to aluminum and gold. This might be explained by the reflectance of those samples. The less light is reflected in the process of white light profilometry, the more likely it is to have singular measurement errors strongly deviating from the real values and raising the uncertainty. This is especially true for the deeper surfaces of pyrolytic graphite.

Neglecting the uncertainty of surface roughness, pyrolytic graphite shows an extraordinary low surface roughness (see Figure 2). It is barely depended on the pulse energy and typically lower than it’s initial value. This is a very desirable property for a laser ablative thruster and could be explained by the strong asymmetry of the crystal structure. This again is the most likely effect generating the rising surface unevenness up to several µm. The authors do expect that a single highly oriented pyrolytic graphite crystal is suitable to compensate this effect and present an ideal MICROLAS propellant.

In the experiments it was observed that with the correct material it is indeed possible to reach ideal parameters. The gold sample while testing the highest pulse energy showed the desired behavior of undetectable changes in surface roughness and unevenness. Due to the microscope images and the EBSD analysis the authors believe the necessary properties to be provided by the microcrystalline structure of the gold. During gold production the lower segment most likely did not introduce as many micro-crystals as the upper segment (compare Figure 4). Microcrystalline structures are known to have a strong effect on the hardness of gold and other materials, which is called Hall-Petch relation [18]. Hardness is not the only effect involved in laser ablative processes and therefore other aspects might even be more relevant as, e.g., the lattice orientation dependent melting behavior at small scales [19]. Nevertheless, the authors expect the microcrystalline property of gold and possibly other metals to be of major relevance to control the evolution of surface unevenness.

## 5. Conclusions and Outlook

For the application of high quality laser ablative thrust generation we require a reproducible surface roughness combined with a low surface unevenness. The separation of the RMS surface roughness into high and low spatial frequency parts shows that the first requirement is given for all conditions investigated. The second requirement of a high evenness appears to be disturbed e.g., by the crystalline structure of the samples. Therefore, it might be desirable to choose from amorphous, mono- or microcrystalline materials with high homogeneity with respect to the laser spot size. One parameter series with gold supports this line of thought. Here the total amount of RMS roughness stays constant within the measurement tolerances while low frequency roughness stays below detection sensibility.

## Supplementary Materials

The following are available online at www.mdpi.com/1996-1944/11/1/50/s1, Figure S1: SEM-scans of aluminum, Figure S2: SEM-scans of copper, Figure S3: SEM-scans of gold, Figure S4: SEM-scans of graphite.

## Nomenclature

$\bar{k}$	highest wavenumber
$\Delta r_{px}$	circle bin size [px]
$\omega_{0}$	beam waist radius
${\bar{r}}_{µm}$	border length [µm]
$\bar{x}$	highest x-position
$\bar{y}$	highest y-position
σ	variance
$F_{\mathrm{peak}}$	peak fluence
NA	numerical aperture
$\underline{k}$	smallest wavenumber
$\underline{x}$	lowest x-position
$\underline{y}$	lowest y-position
H(x,y)	2-dimensional Hanning window
$n_{x,y}$	number of pixels in x,y direction
$r_{px}$	circle pixel radius
*S*	scaling factor
EBSD	Electron backscattered diffraction
f	focal length
FFT	fast Fourier transformation
HF	high frequency
LF	low frequency
PSD	power spectral density
RMS	root mean square
ROI	region of interest
*x*,*y*	*x*,*y*-coordinate
