# Supplementary material for: Power Spectral Density Evaluation of Laser Milled Surfaces
Source: Materials (Basel). 2017 Dec 29;11(1):50. doi: 10.3390/ma11010050 (PMC5793548; doi:10.3390/ma11010050)
Supplement: Supplementary file 1 [file materials-11-00050-s001.pdf]

materials

MDPI

Addendum

Supplementary Figures to: "Power Spectral Density evaluation of laser milled surfaces"

Raoul-Amadeus Lorbeer [1]*, Jan Pastow [1,2], Michael Sawannia [1], Peter Klinkenberg [1], Daniel Johannes Förster [2] and Hans-Albert Eckel [1]

1 German Aerospace Center (DLR), Institute of Technical Physics, Pfaffenwaldring 38-40, 70569 Stuttgart, Germany
2 Institut für Strahlwerkzeuge, University of Stuttgart, Pfaffenwaldring 43, 70569 Stuttgart, Germany
* Correspondence: Raoul.Lorbeer@dlr.de; Tel.: +49-711-6862-8263

Received: 20 November 2017; Accepted: 22 December 2017; Published: 29 December 2017

Abstract: These figures were partially extracted from the master thesis "Optimierung eines Versuchsaufbaus und Untersuchungen des Materialabtrags für Laser-ablative Mikroantriebe" by Jan Pastow at the Institut für Strahlwerkzeuge, University of Stuttgart, Pfaffenwaldring 43, 70569 Stuttgart, Germany and translated to English language. All fluences are calculated as peak fluences.

Keywords: surface roughness; surface unevenness; laser milling; power spectral density; micro crystalline; Hall-Petch; EBSD

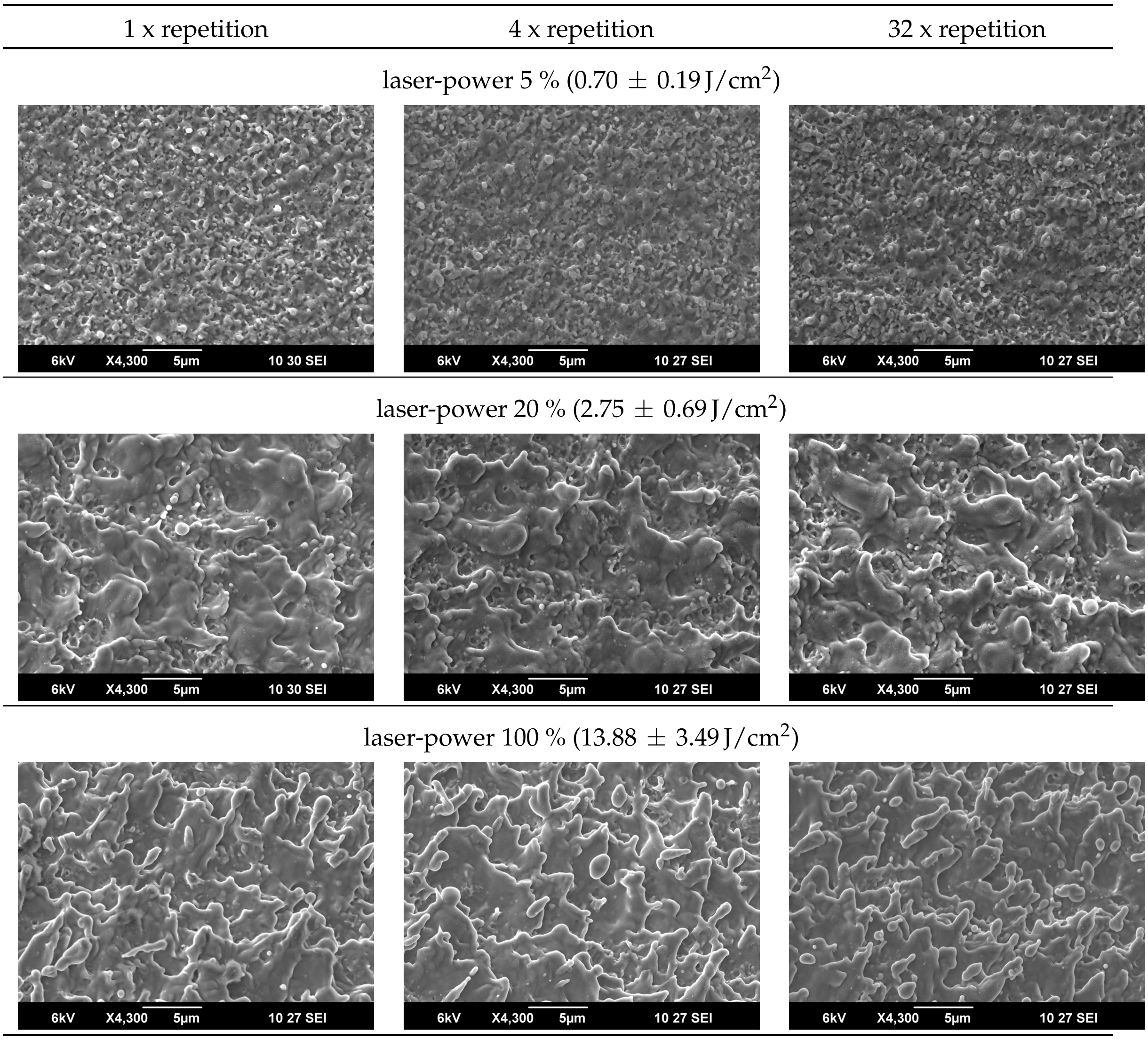

Figure S1. SEM-scans of aluminum for 1x, 4x, 32x repetitions and 5%, 20%, 100% laser-power.

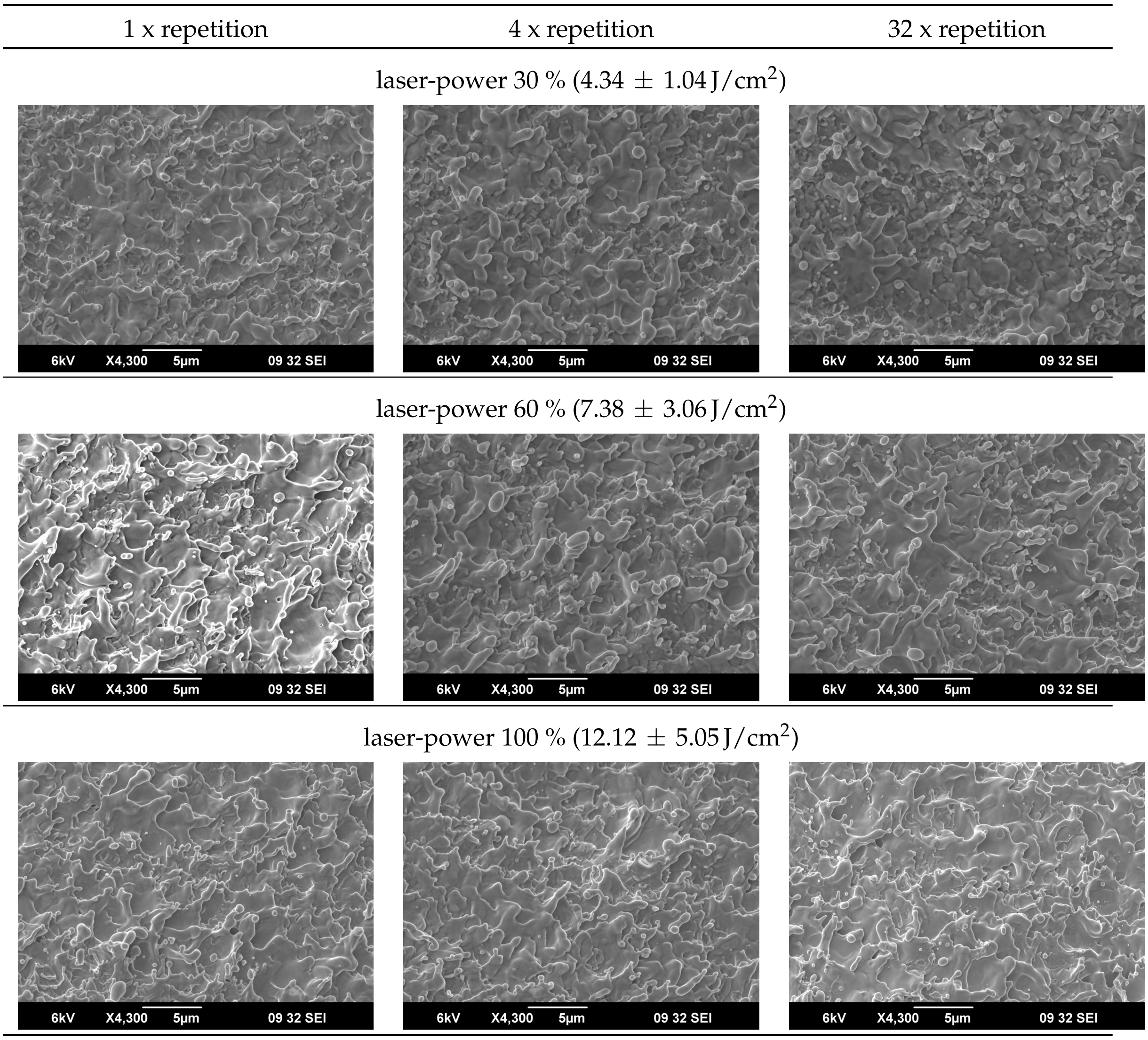

Figure S2. SEM-scans of copper for 1x, 4x, 32x repetitions and 30%, 60%, 100% laser-power.

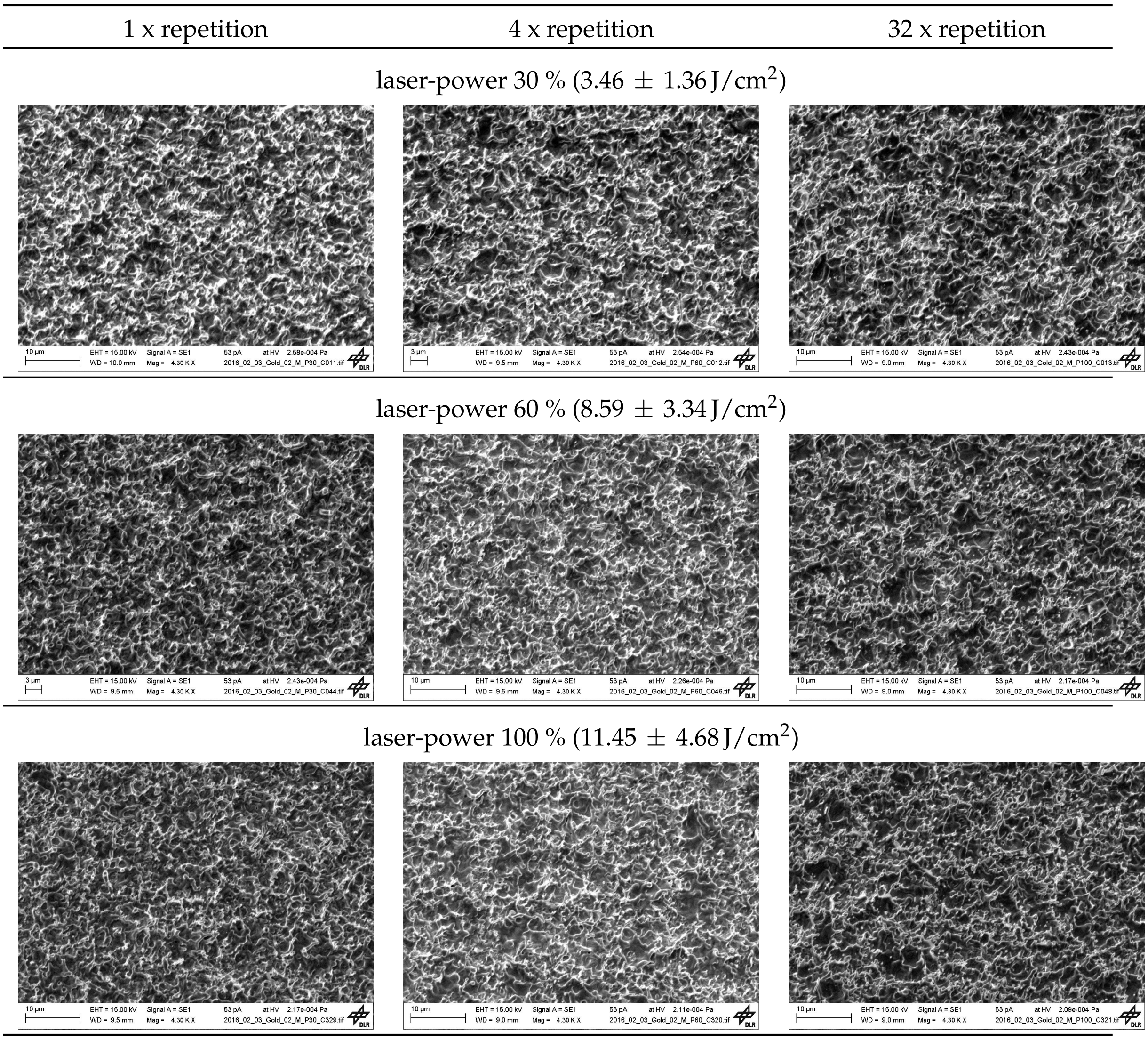

Figure S3. SEM-scans of gold for 1x, 4x, 32x repetitions and 30%, 60%, 100% laser-power.

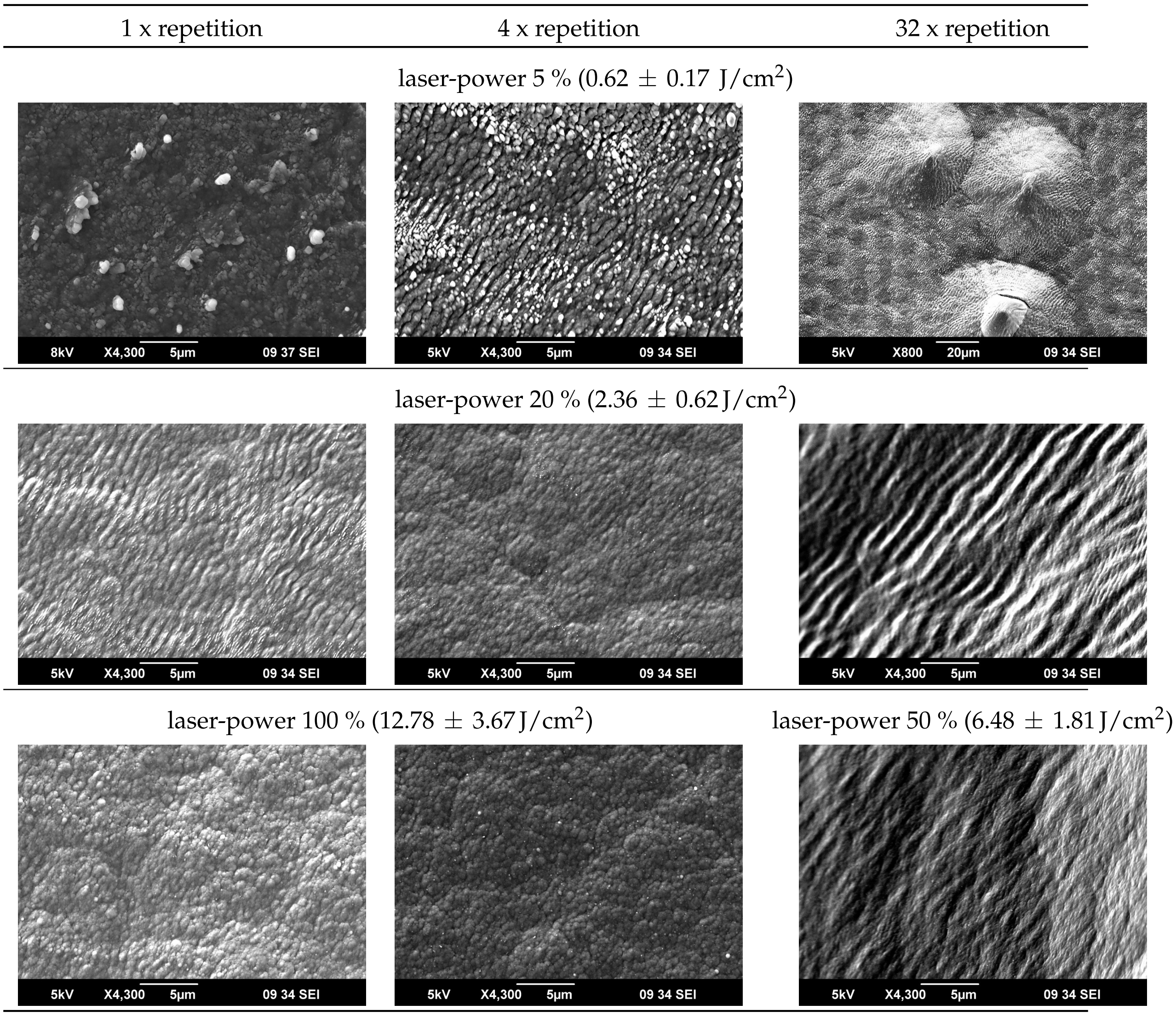

Figure S4. SEM-scans of graphite for 1x, 4x, 32x repetitions and 5%, 20%, 100%(50%) laser-power.

© 2018 by the authors. Licensee MDPI, Basel, Switzerland. This article is an open access article distributed under the terms and conditions of the Creative Commons Attribution (CC BY) license (http://creativecommons.org/licenses/by/4.0/).
